# Case report: Molecular analysis of a 47,XY,+21/46,XX chimera using SNP microarray and review of literature

**DOI:** 10.3389/fgene.2022.802362

**Published:** 2022-11-11

**Authors:** Chariyawan Charalsawadi, Somchit Jaruratanasirikul, Areerat Hnoonual, Aussanai Chantarapong, Pornsiri Sangmanee, Sasipong Trongnit, Natini Jinawath, Pornprot Limprasert

**Affiliations:** ^1^ Department of Pathology, Faculty of Medicine, Prince of Songkla University, Songkhla, Thailand; ^2^ Genomic Medicine Center, Faculty of Medicine, Prince of Songkla University, Songkhla, Thailand; ^3^ Department of Pediatrics, Faculty of Medicine, Prince of Songkla University, Songkhla, Thailand; ^4^ Program in Translational Medicine, Faculty of Medicine Ramathibodi Hospital, Mahidol University, Bangkok, Thailand; ^5^ Chakri Naruebodindra Medical Institute, Faculty of Medicine Ramathibodi Hospital, Mahidol University, Samut Prakan, Thailand; ^6^ Integrative Computational Bioscience Center (ICBS), Mahidol University, Nakhon Pathom, Thailand

**Keywords:** chimerism, mosaicism, down syndrome, mechanism, SNP microarray

## Abstract

Chimerism is a very rare genetic finding in human. Most reported cases have a chi 46,XX/46,XY karyotype. Only three non-twin cases carrying both trisomy 21 and a normal karyotype have been reported, including two cases with a chi 47,XY,+21/46,XX karyotype and a case with a chi 47,XX,+21/46,XY karyotype. Herein we describe an additional case with a chi 47,XY,+21/46,XX karyotype. For the case, a physical examination at the age of 1 year revealed ambiguous genitalia with no features of Down syndrome or other malformations. Growth and developmental milestones were within normal ranges. We performed short tandem repeat (STR) and single nucleotide polymorphism (SNP) microarray analyses to attempt to identify the mechanism underlying the chimerism in this patient and the origin of the extra chromosome 21. Cytogenetic analyses of the patient’s peripheral blood revealed approximately 17% of a 47,XY,+21 lineage by G-banding karyotype analysis, 13%–17% by FISH analyses of uncultured peripheral blood, and 10%–15% by SNP microarray analysis. Four years later, the percentage of trisomy 21 cells had decreased to approximately 6%. SNP microarray and STR analyses revealed a single maternal and double paternal genetic contribution to the patient for the majority of the markers, including the chromosome 21 markers. The extra chromosome 21 was paternally derived and meiosis I nondisjunction likely occurred during spermatogenesis. The mechanisms underlying chimera in our case was likely fertilization two spermatozoa, one with an ovum and the other with the second polar body.

## Introduction

Chimerism and mosaicism are rare phenomena in human. The difference between chimerism and mosaicism depends on the number of zygotes involved in the developmental process. While mosaicism results from a mitotic error in a single zygote, chimerism develops from the fusion of two or more separate zygotes. Chimerism has rarely been reported and is far less common than mosaicism. Acquired chimerism occurs later in life through transfusion or transplantation, while constitutional chimerism is a condition that an individual is born with. The latter can be categorized into two groups, partial chimerism and whole-body chimerism. Partial chimerism results from feto-fetal transfusion between dizygotic twins (twin chimera) or feto-maternal transplacental exchange (microchimera), while whole-body chimerism arises as the result of the union of two or more different zygotes into one body (fusion chimera) (Sawai et al., 1994; Boklage, 2006; Madan, 2020). Most chimeras remain undetected, and thus the actual incidence is unknown. Chimeras of the same sex would have a normal phenotype and thus are usually only discovered by chance, such as through paternity testing, blood group testing, or tissue donor testing prior to organ transplantation. Most chimeras are detected because of the coexistence of XX and XY chromosomal complements in an individual who has ambiguous genitalia. However, the phenotypic manifestations of chimeras vary, ranging from normal female or male genitalia to variable degrees of genital ambiguity.

Several mechanisms underlying whole body chimerism have been proposed, such as 1) tetragametic chimera formed by double fertilization followed by an early fusion of dizygotic twins, 2) tetragametic chimera formed through double fertilization of an ovum and a second polar body and subsequent fusion of the zygotes, 3) parthenogenetic chimera formed by fertilization of two daughter cells derived from parthenogenetic division of the female pronucleus (two identical gametes from parthenogenetic activation) with two spermatozoa, 4) gynogenetic chimera formed by parthenogenetic division of female pronucleus, then one daughter cell fertilized by a spermatozoa and diploidization of the other daughter cell, 5) androgenetic chimera formed by fertilization of an ovum with two spermatozoa and subsequent diploidization of triploid zygotes (Sheppard et al., 2019; Kawamura et al., 2020; Madan, 2020). Among chimera cases that were analyzed by molecular methods, tetragametic chimera was by far the most common mechanism (Reviewed in Medan 2020). Parthenogenetic chimeras have been reported and it is somewhat difficult to distinguish these from chimera that polar body fertilization is involved. A parthenogenetically-activated ovum arises from an ovum undergoing endoreplication forming a diploid ovum that develops without fertilization into a female zygote (Malan et al., 2006; Winberg et al., 2010; Minelli et al., 2011). Theoretically, parthenogenetic chimera would have 2 cell lines with genotype similarity more than in chimera that polar body fertilization is involved.

Only a few individuals with chimerism with an abnormal cell lineage coexisting with a normal lineage have been reported. Chimeras with trisomy 21 and diploid cell lines have been reported in both twin pregnancies (Hwa et al., 2006; Lucon et al., 2006; Bogdanova et al., 2010) and singleton pregnancies (Sawai et al., 1994; Ramsay et al., 2009; Lee et al., 2012). Only two cases of chimeras with trisomy 21 and another cell line with a different abnormality have been reported (Wiley et al., 2002; Vorsanova et al., 2006). Herein, we describe the clinical findings and molecular analyses of a new case of chi 47,XY,+21/46,XX. In this case, we studied the mechanism of chimerism formation by using short tandem repeat (STR) and single nucleotide polymorphism (SNP) array analyses.

### Clinical presentation

The patient was born with a weight of 3,670 g (90th-97th percentile), length of 54 cm (>97th percentile), and head circumference of 33 cm (25th–50th percentile). The patient was an only child born to healthy non consanguineous parents. Maternal age at birth of the patient was 25 years old. A transvaginal ultrasonography at gestational age of 8 weeks showed a singleton pregnancy. The course of pregnancy was uneventful; however, the patient was delivered through caesarian section due to fetal distress at 41 weeks of gestation. Ambiguous external genitalia were noted at birth. A physical examination at 1 month found a small phallus, a urogenital opening, labioscrotal swellings without rugae formation, and a palpable gonad in the right inguinal area. Other than the ambiguous genitalia there were no other obvious physical abnormalities, including no clinical manifestations of Down syndrome. After a family meeting with a neonatologist, geneticist and pediatric endocrinologist, the parents decided to raise the child as a girl since the external genitalia were predominately female and female was entered on the birth certificate. After that the patient was followed-up yearly, with the most recent follow-up at 4 years of age. At that time the parents said that the patient dressed as a girl and liked playing with dolls and a cooking set. Her physical growth was average with height and weight along the 50th percentiles for Thai girls. Her developmental milestones were assessed to be normal for age. She enjoyed going to kindergarten and played in a group with many other girls. Her parents were satisfied with her sex assignment as a girl since there had been no progression in phallus length and her genitalia looked more like a girl. The parents decided to postpone any decisions regarding an orchidectomy or genitoplasty until the patient reached adolescence and could make her own decision to be male or female at which time surgical corrections would be done.

## Materials and methods

### Karyotype and fluorescence *in situ* hybridization analyses

Double lymphocyte cultures were established according to standard synchronization procedures. A total of 95 metaphase spreads were counted and five metaphase spreads analyzed following GTG-banding. We carried out fluorescent *in situ* hybridization (FISH) analysis on uncultured lymphocytes. Two probe sets were used, a set specific to chromosome 21 (21q22.13-22.2, Spectrum Orange) and chromosome X (Xp11.1-q11.1, Spectrum Green) and a set specific to the sex-determining region Y (SRY) gene (Yp11.3, Spectrum Orange) and chromosome X (Xp11.1-q11.1, Spectrum Green) (Vysis; Abbott Molecular, United States). The fluorescent signals were visualized using a Metafer Scanning and Imaging Platform and ISIS system (MetaSystems, Germany). A thousand interphase cells were spot counted with each probe set.

### Single nucleotide polymorphism microarray analysis

DNA derived from uncultured peripheral blood samples of the patient and parents were analyzed by SNP microarray, according to the manufacturer’s protocol (Illumina, United States). We used an Illumina HumanCytoSNP-12 array, BlueFuse Multi software, and GenomeStudio software v.2.0.3 (Illumina, United States), which allowed whole-genome analysis of ∼ 300,000 SNPs across the genome. Log R ratio and B-allele frequency were calculated. Trio analysis was done using SNP graphs created by the GenomeStudio software to determine parental contributions to each of the informative SNPs. The patient’s SNP loci with no-call (NC) result were manually inspected when a heterozygous allele presented in one parent and a homozygous allele presented in the other parent. In such cases, a paternal contribution to the allele was deemed when the mother had a homozygous allele (AA) and the father had a heterozygous allele (AB), and we then assigned the patient’s NC allele as AAAB, and a maternal contribution was deemed for the opposite situation.

### Short tandem repeat analysis

DNA samples were extracted from peripheral blood of the patient and parents using a FlexiGene ^®^ DNA kit (Qiagen, United States) following the manufacturer’s protocol. DNA samples were analyzed using an AmpFLSTR™ Identifiler™ Plus PCR Amplification Kit and an ABI3130 Genetic Analyzer (Applied Biosystems, United States) according to the manufacturers’ protocols. A total of 20 genetic markers were analyzed using the GeneMapper^®^ ID Software Version 3.2.1 (Applied Biosystems, United States). We calculated allelic ratios of the patient and her parents from the area under the curve derived from the same software.

To determine the origin of the extra chromosome 21, a quantitative fluorescent PCR test for rapid aneuploidy detection was performed using the Devyser Compactv3 QF-PCR Kit (QF-PCR; Devyser Compact v3, Devyser, Stockholm, Sweden), and the Devyser Resolution 21 v2 kit (D21S1435, D21S11, D21S1411, D21S1444, D21S1442, D21S1437, D21S2055, D21S1409, D21S1280), and according to the manufacturer’s instructions. Additionally, ten additional STR markers (D21S408, D21S411, D21S236, D21S120, D21S415, D21S369, D21S1264, D21S1414, D211440, and D21S2055) were used to increase the possibility of detecting trisomy 21 chimera in our case. Fluorescence PCR was performed using primers labeled by FAM and VIC. The details of the STR markers and primers are shown in Supplementary Table S1. Each STR marker was amplified by PCR of genomic DNA isolated from peripheral blood. In addition, PCR was performed on DNA isolated from buccal cells. All PCR reactions were carried out in a reaction volume of 10 μl containing 25–50 ng genomic DNA, 1.5 mM MgCl_2_, 0.2 mM dNTP, 0.2 μM each of fluorescent-labeled forward primer and reverse primer, 1X PCR buffer and 0.2 units of IMMOLASE™ DNA polymerase. The PCR conditions were 35 cycles of an initial denaturing step for 10 min at 95°C; a denaturing step for 1 min at 95°C; an annealing step for 1 min at 55°C; an extension step for 1 min at 72°C; and a final extension step for 10 min at 72°C. The amplified DNA samples were separated by electrophoresis using an ABI 3500 Genetic Analyzer, and the analysis of each allele for specific markers to identify their sizes was performed using GeneMapper Software ver. 5.0 (Applied Biosystems, Waltham, MA, United States). Trisomy 21 was detected, with the correspondent specific STR markers, either as three peaks of fluorescent signal or as two unbalanced peaks with an area ratio of 2:1.

## Results

### Karyotype and fluorescence *in situ* hybridization analyses analyses

**FIGURE 1:**
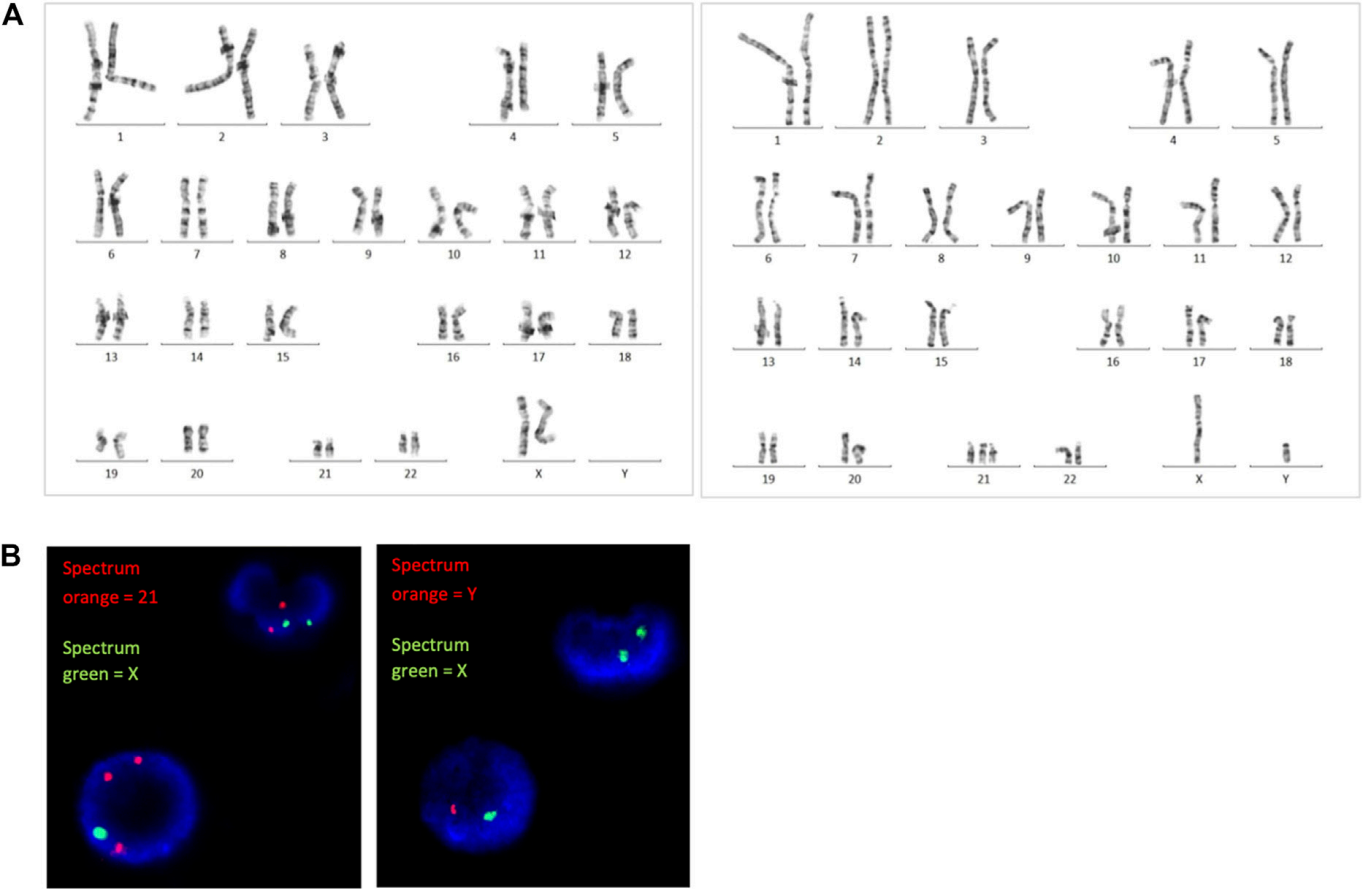
**(A)** G-banding karyotype analysis and **(B)** FISH analysis revealed the presence of both trisomy 21 and diploid cells.

GTG-banding chromosome analysis revealed 2 cell lines, 46,XX and 47,XY,+21. The ratio of the 46,XX to 47,XY,+21 karyotypes was 83:17 (i.e., the percentage of trisomic cells was 17%). FISH analysis on uncultured peripheral blood revealed approximately 12.8% of trisomy 21 cells using a set of probes specific to chromosomes 21 and X, and 17.4% of XY cells using a set of probes specific to chromosomes Y and X (Figure 1).

### Single nucleotide polymorphism microarray analysis

**FIGURE 2:**
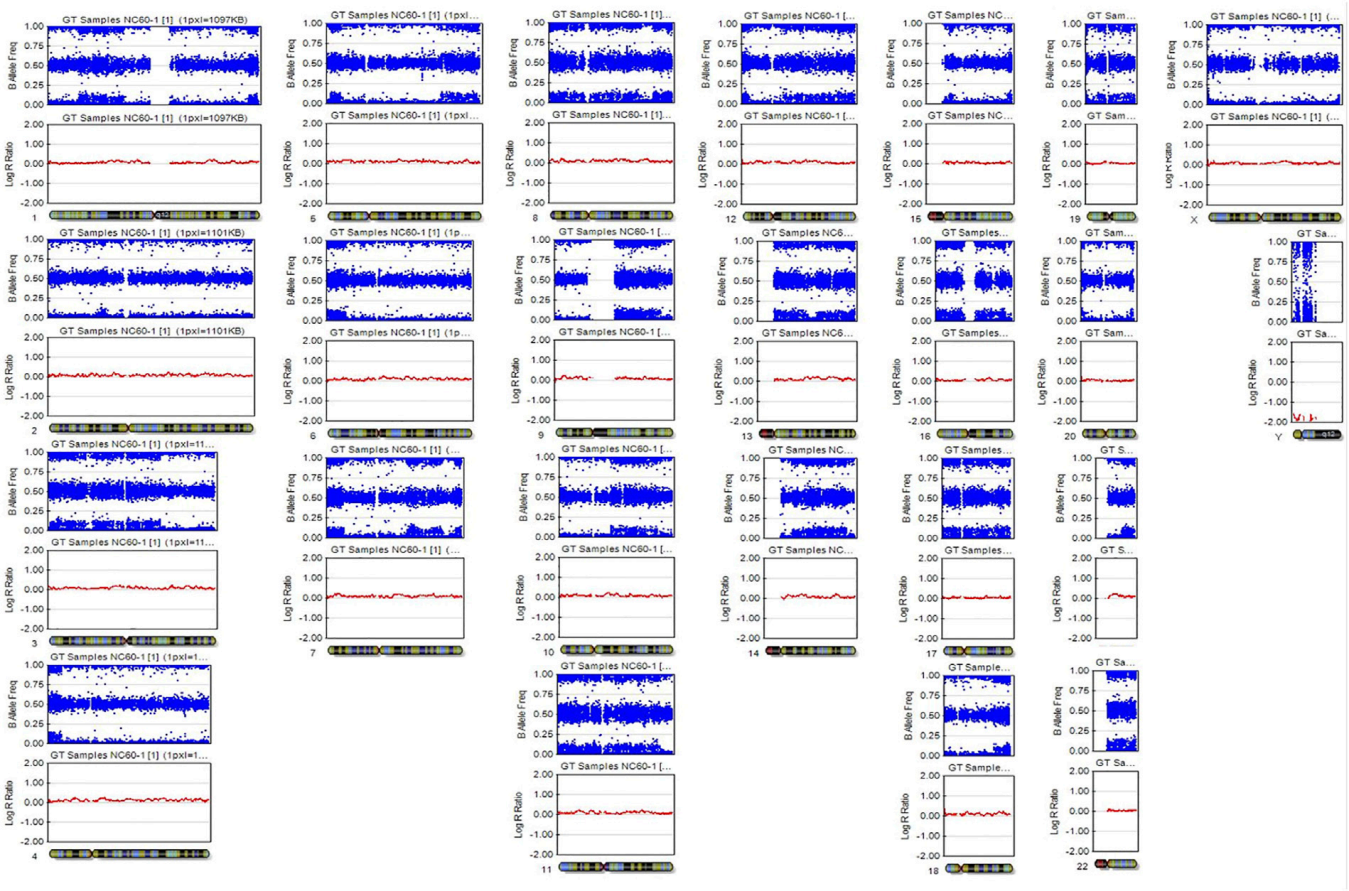
SNP microarray demonstrating a patchy pattern of B-allele frequencies across the genome, which is shown as altered allelic combinations from five tracks to three tracks.

**FIGURE 3:**
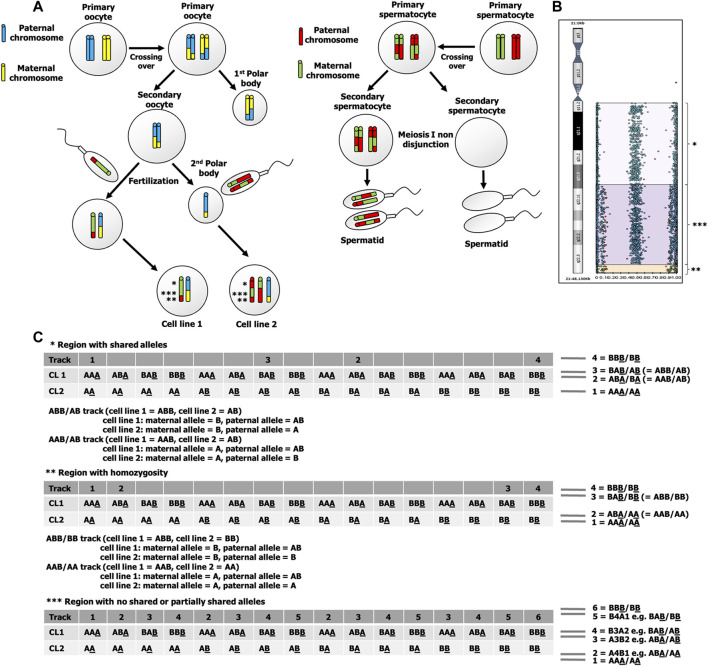
**(A)** Fertilization of two spermatozoa, one with an ovum and the other with the second polar body. This underlying mechanism results in 2 cell lines that has three different allelic patterns, indicated by *, **, and ***. **(B)** B-allele frequency graph of chromosome 21 shows altered allelic combinations from four tracks (BBB/BB, BAB/AB, ABA/BA, and AAA/AA) to six tracks (BBB/BB, B4A1, B3A2, A3B2, A4B1, and AAA/AA). The four-track pattern at a region near centromere of the long arm of chromosome 21 indicates a shared allelic pattern between cell line 1 and cell line 2, while six-track pattern indicates a no shared or partially shared allelic pattern between cell line 1 and cell line 2. At a region near telomere of the long arm of chromosome 21, there are four tracks with a pattern of homozygosity (BBB/BB, BAB/BB, ABA/AA, and AAA/AA). **(C)** Possible genotypes of cell line 1 and cell line 2 of three regions of chromosome 21 that are indicated by *, **, and ***. The underlined allele indicates a maternal allele, CL1 indicates cell line 1, and CL2 indicates cell line 2.

**FIGURE 4:**
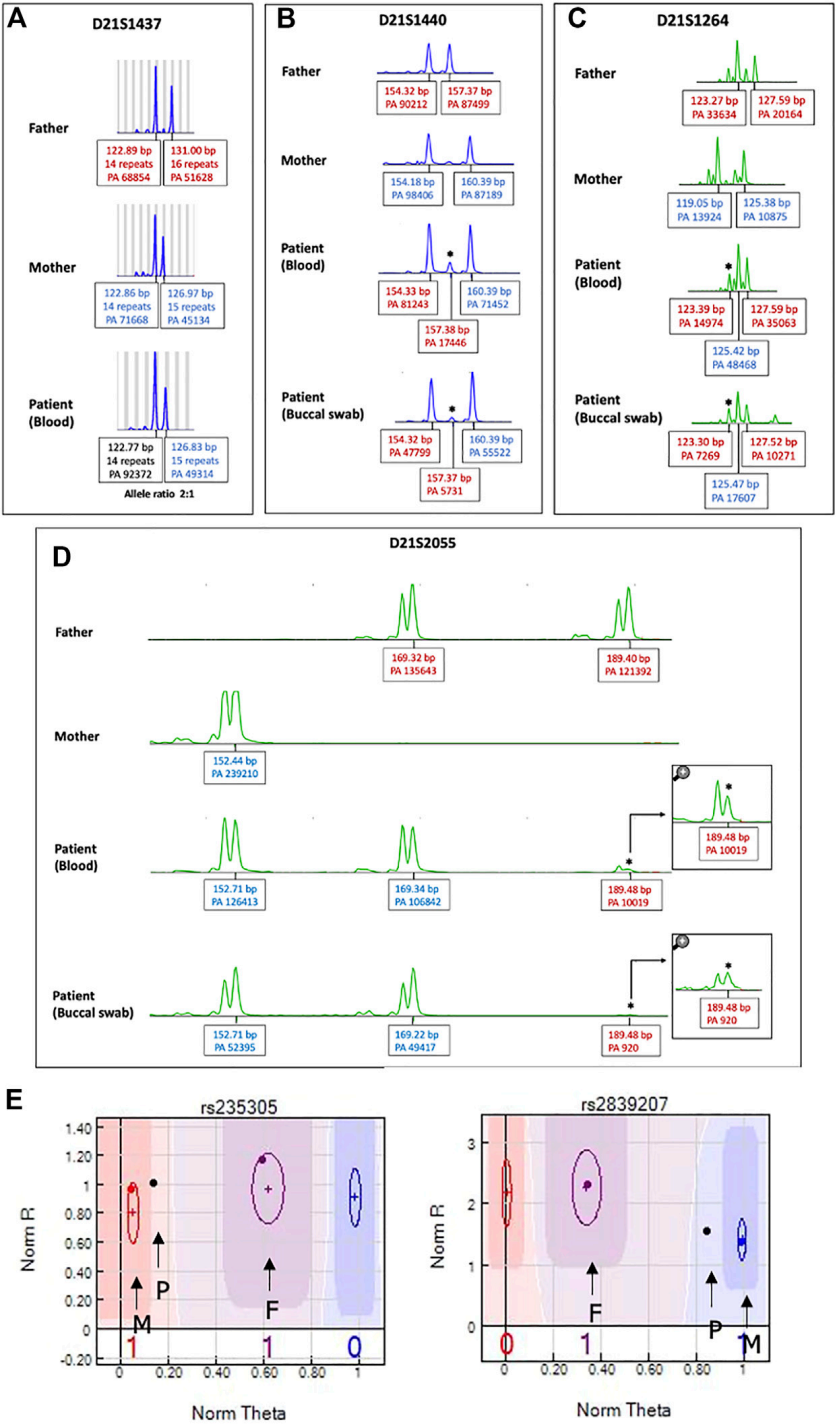
STR analysis of chromosome 21 to identify origin of the extra chromosome 21. Patient’s DNA obtained from both peripheral blood and buccal cells. The extra chromosome was detected either as **(A)** a skewed 2:1 ratio in one chromosome 21 marker (D211437) or **(B–D)** a small extra allele for three markers (D21S1264, D21S1440, D21S2055), as indicated by asterisks. PA: peak area, bp: base pair. **(E)** SNP trio analysis of chromosome 21 SNP markers. An SNP graph at rs235305 showing a homozygous allele (AA) contributed from the mother and a heterozygous allele (AB) contributed from the father. Thus, the patient’s “no-call” genotype was assigned as AAAB. A SNP graph at rs2839207 showing a homozygous allele (BB) contributed from the mother and a heterozygous allele (AB) contributed from the father. Thus, the patient’s “no call” genotype was assigned as ABBB. P indicates the SNP of the patient, F indicates the SNP of the father, and M indicates the SNP of the mother.

A SNP microarray revealed a generalized patchy pattern of B-allele frequencies across the genome. The percentage of trisomy 21 cells estimated from the SNP microarray was 10%–15%. Altered allelic combinations on the same chromosome were seen as the patchy patterns. Because the patient had both XX and XY cell lines, five allelic combinations for each SNP locus (AAAA, AAAB, AABB, ABBB, and BBBB) were possible (Figure 2). We also detected a pattern of homozygosity near telomere of the long arm of chromosome 21 (Figure 3). SNP trio analysis revealed that almost all of the SNP loci in the patient had homozygous (either AA or BB) alleles contributed from the mother and a heterozygous (AB) allele contributed from the father for the entire genome, including chromosome 21 (Figure 4E, Supplementary Table S4). However, a heterozygous allele contributed from the mother and homozygous alleles contributed from the father were detected in a few loci.

### Short tandem repeat analysis

Using a human identification kit with 20 genetic markers showed both paternal and maternal allele contributions to the patient. Three alleles at seven different loci were detected. Of these, five loci indicated a double paternal contribution and the other two loci indicated a double maternal contribution to the patient (Supplementary Table S2). However, the origin of the extra chromosome 21 in this case could not be determined by STR analysis as there were limited numbers of chromosome 21 markers and all markers on chromosome 21 were noninformative. Therefore, we extended the STR analysis for chromosome 21 using 19 additional STR markers. Three markers (D21S1264, D21S1440, D21S2055) showed small extra alleles with double paternal contributions (Figures 4B–D), while one marker (D21S1437) showed a slightly skewed allele ratio, which was inconclusive (Figure 4A). All other informative markers showed allele ratios of 1:1 (Supplementary Table S3).

We evaluated the ability of STR analysis using fluorescent PCR with capillary electrophoresis to detect low-level mosaicism/chimerism for trisomy 21. As recurrent stutters (i.e., not true alleles) may prevent the resolution of the STR loci, we distinguished stutter peaks for true alleles at the threshold of detection of multiplex STR typing by making a calibration curve of a DNA mixture representing different proportions of aneuploid cells. We mixed DNA obtained from an individual with a normal chromosomal complement and DNA obtained from an individual with full trisomy 21 at different ratios with the total genomic DNA input maintained at 50 ng. Four artificial mosaic DNA samples were generated to mimic 10%, 20%, 30%, and 50% mosaicism for trisomy 21. STR analysis of the artificial sample with 50% mosaic trisomy 21 clearly showed two patterns, either three peaks of chromosome 21 or two unbalanced peaks with an area ratio of 2:1. For the artificial samples with 20% and 30% mosaic trisomy 21, STR analysis showed smaller peaks similar to our chimeric case. For the sample with 10% mosaic trisomy 21, we were unable to detect any peaks indicating a trisomic allele (data not shown). STR analysis using DNA obtained from buccal cells resulted in the same findings as the DNA obtained from peripheral blood.

## Discussion

A chimera is defined as an individual with genetically different cell lineages developed from two or more zygotes. This is different from a mosaic, which is defined as the presence of two or more genetically different cell lineages in an individual that has developed from a single fertilized egg (Boklage, 2006; Gardner et al., 2011). Several mechanisms underlying whole body chimerism have been proposed. In addition, it has been hypothesized that chimerism may actually arise from mosaicism, for example, a mosaic XX/XY that arose from an XXY zygote with subsequent two non-disjunction events (Niu et al., 2002). Detailed molecular analyses using a large number of genetic markers can help distinguish between these two conditions, in which mosaicism should have one maternal and one paternal allelic contribution (Malan et al., 2006). Mosaicism was very unlikely in our case as post-zygotic nondisjunctional errors would have had to occur to result in chromosomes X, Y, and 21 in a 48,XXY,+21 zygote.

Chimeras with an abnormal cell lineage coexisting with a normal one are extremely rare. Multiple congenital anomalies have been reported in a chimera with 47,XY,+21/47,XX,+12 (Wiley et al., 2002), although this case may have resulted from a co-occurrence of two trisomies. Previous case reports of chimeras with trisomy 21 lineage and their phenotypic findings are reviewed in Table 1. No clinical features of Down syndrome were observed in our case, similar to that of individuals in other studies (Table 1). It is difficult to draw a solid conclusion with only such a small number of reported cases available; however, it is interesting to note that none of the chimeras born in singleton pregnancies exhibited obvious or notable Down syndrome phenotypes, while chimeras born in twin pregnancies had highly variable phenotypic manifestations, ranging from normal phenotypes to typical features of Down syndrome or multiple congenital anomalies.

**TABLE 1:** Case reports of chimera with trisomy 21 and normal cell lineages.

Reference	Type of analyzed cells	Karyotype	%Trisomic cells (%)	Clinical report	Mechanism of chimerism
**Twin pregnancy**					
Hwa et al. (2006)	Lymphocyte	47,XX,+21 [3]/46,XX [35]/46,XY [7]	7	Normal post-mortem findings	Transfer of XY cell line from twin B to twin A
				Prenatal sonography showed 2 amniotic sacs, no visible connection between the two placentae, and no signs of twin-twin transfusion syndrome	
				The co-twin had 46,XY and normal post-mortem findings	
Lucon et al. (2006)	Lymphocyte	47,XY,+21 [5]/46,XX [30]	14	Presence of multiple malformations, including anencephaly, cervical rachischisis, absence of neck, submucous cleft palate, trunk-limb disproportion, thoracic scoliosis, thoracic hemivertebrae, 11 ribs on the right and 9 ribs with fusion on the left, camptodactyly, clinodactyly of the 5th fingers, prominent calcaneus, absence of left kidney, and uterus unicornis	Fusion of two zygotes
				Prenatal sonography showed monochorionic diamniotic placenta	
				The co-twin had 46,XX and was clinically normal	
Bogdanova et al. (2010)	Lymphocyte	XX 3 cells, XY 97 cells	97	Presence of typical features of Down syndrome. Normal female external genitalia, aplasia of uterus and fallopian tubes, and gonads suggestive of ovarian structures	Feto-fetal transfusion
	Skin fibroblast	47,XX,+21	100	Prenatal sonography showed monochorionic diamniotic placenta	
				Feto-fetal transfusion from co-twin brother, who was 46,XY and was clinically normal	
**Singleton pregnancy**					
Sawai et al. (1994)	Lymphocyte	47,XY,+21 [6]/46,XX [294]	2	No clinical features of Down syndrome	Early fusion of two different zygotes, one with a normal 46,XX and the other with 47,XY,+21 karyotype or from double fertilization of an ovum or a polar body by two different spermatozoa.
	Skin fibroblast	47,XY,+21 [7]/46,XX [178]	4	Ambiguous external genitalia with small phallus, perineal hypospadias, bifid scrotum containing gonads, vagina with no external vaginal orifice	
Ramsay et al. (2009)	Lymphocyte	47,XY,+21 [10]/46,XX [90]	10	No obvious features of Down syndrome, except for a transverse crease on the right hand	Postzygotic fusion of two independent zygotes
	Left gonad	47,XY,+21 [11]/46,XX [51]	18	Ambiguous genitalia with very small phallus with hypospadias on the ventral surface, and scrotal sacs with two palpable gonads. Gonad biopsy revealed ovotestis on left gonad and testis on right gonad (true hermaphrodite)	
	Right gonad	47,XY,+21 [31]/46,XX [23]	57	Normal developmental milestones. Presence of hypopigmented patches on skin	
Lee et al. (2012)	Cortex and placenta	47,XX,+21 [9]/46,XY [11]	45	No obvious features of Down syndrome except for an enlarged gap between 1st and 2nd toes of both feet.	Parthenogenetically activated ovum fertilized with two spermatozoa with opposite sex chromosomes
	Skin and kidney	47,XY,+21 [5]/46,XX [11]	29	Normal male development of external genitalia and gonads except for bilateral cryptorchidism	
Our case	Lymphocyte	47,XY,+21 [17]/46,XX [83]	17	No clinical features of Down syndrome	Fertilization of an ovum and the second polar body by two spermatozoa
				Ambiguous genitalia with urogenital opening, small phallus, labioscrotal swelling without rugae formation, and palpable gonad on the right inguinal area	
				Normal developmental milestones	

Various possible genetic mechanisms underlying chimera with trisomy 21 have been suggested (Sawai et al., 1994; Ramsay et al., 2009; Lee et al., 2012). The presence of two alleles from the mother and two alleles from the father for chromosome 21 loci and other autosomal loci was identified in one patient in a 2009 study, suggesting a mechanism of two spermatozoa fertilizing two independent ova (Ramsay et al., 2009). In another study, the patient had one allele from the mother and two alleles from the father, with the additional chromosome 21 originating from the father, suggesting fertilization of two spermatozoa, one with chromosome Y and the other with chromosome X,+21, and a parthenogenetically activated ovum (Lee et al., 2012). Fertilization of a parthenogenetic activated ovum by two spermatozoa has also been described in two cases with 46,XX/46,XY (Giltay et al., 1998; Shin et al., 2012), and in one case with a 46,XX/47,XY,+14 karyotype (Winberg et al., 2010).

In our case, the findings from STR analysis using a human identification kit with 20 genetic markers showed both double paternal contributions and a double maternal contribution to the patient in seven different informative loci suggests that chimerism may arise from early fusion of dizygotic twins from double fertilization (tetragametic chimera). However, dispermic fertilization of a parthenogenetically activated ovum or double fertilization of an ovum and a polar body by two different spermatozoa with subsequent fusion of the zygotes could not be ruled out in our case as STR analysis used only a limited number of STR markers. Also, interpretation of an STR analysis to determine the contribution of the paternal versus maternal alleles to the offspring genotype profile as bleeps of fluorescent peaks is challenging, especially in cases of low-level mosaicism/chimerism. When the percentage of one cell line is much lower than that of the other cell line as in our case, the percentage of XY cells with trisomy 21 was approximately 10%–15%, although the default threshold value could be adjusted to bring up the same allele sizes as present in the parents. A better way to distinguish stutter peaks (i.e., not true alleles) for true alleles is to make a calibration curve. We made a calibration curve of a DNA mixture representing different proportions of trisomy 21 cells. By doing this, we were able to clearly determine the contribution of two paternal alleles to the offspring genotype profile.

The SNP microarray has provided useful information for identification of mechanisms underlying chimera in previous studies (Conlin et al., 2010; Shin et al., 2012; Bottega et al., 2019). As it has more genetic markers, the SNP microarray has a greater advantage for research relating to the underlying mechanisms of mosaicism and chimerism. Main mechanisms underlying chimerism and expected SNP microarray results are depicted in Supplementary Figure S1. Based on the findings of homozygous maternal allele (AA or AB) and heterozygous paternal allele (AB) in almost all informative SNP loci in our patient, this leads to the probability that the underlying mechanism in our case was either fertilization of the ovum and the second polar body by two spermatozoa or fertilization of two daughter cells of a parthenogenetically activated ovum by two spermatozoa. In our case, we found that the extra chromosome 21 was paternally derived. To determine whether fertilization of the polar body or fertilization of a parthenogenetically-activated ovum was involved in the underlying mechanism is to search for evidence of crossing over. At near centromere of chromosome 21, there are four tracks of B-allele frequency graph that showed a pattern of shared alleles between 2 cell lines. Since a single maternal and double paternal genetic contribution to the patient for the majority of the markers, the maternal allele of both cell lines must be the same, for example, if the maternal allele of cell line 1 is “A”, the maternal allele of cell line 2 is also “A”. As there are two heterozygous tracks (“AB” and “BA”) in cell line 2, it indicates that the maternal allele and the paternal allele are different, for example, the maternal allele is “A”, the paternal allele is “B”. When both cell lines are considered (“cell line one genotype/cell line 2 genotype”), there are two heterozygous tracks (“ABB/AB” and “AAB/AB”). As the maternal alleles of both cell lines are the same, therefore, the paternal allele must be heterozygous (“AB”) in cell line 1. At near telomere of chromosome 21, there are four tracks of B-allele frequency graph that showed a pattern of homozygosity. As there are two homozygous tracks (“ABA/AA” and “BAB/BB”), when cell line 2 is homozygous (“AA” or “BB”) and the maternal alleles of both cell lines are the same, therefore, the paternal allele of cell line 1 is heterozygous “AB” (Figure 3). As the paternal allele of cell line 1 can be heterozygous and crossing over tends not to occur at a region of near centromere, it is likely meiosis I nondisjunction occurred during spermatogenesis. The maternal alleles of two cell lines in our case can be different in a region with no shared paternal and maternal allele, which exclude the possibility of fertilization of a parthenogenetically activated ovum. This finding is consistent with the finding of double maternal and single paternal contribution to the patient genotype in a few STR loci and SNP loci (Supplementary Table S4).

In conclusion, to date a large proportion of human chimeras have not undergone detailed genetic analysis. It is necessary to collect more information regarding the clinical details and origins of chimerism, which will provide more information for analysis into the mechanisms underlying fertilization errors. In addition, clinical data of chimeras with abnormal cell lineages, such as trisomy 21, need to be collected. This information will be useful for genetic counselling and decision making, especially in the prenatal diagnosis setting.

## Data Availability

The datasets for this article are not publicly available due to concerns regarding participant/patient anonymity. Requests to access the datasets should be directed to the corresponding author.
