# Supplementary material for: Case report: Molecular analysis of a 47,XY,+21/46,XX chimera using SNP microarray and review of literature
Source: Front Genet. 2022 Nov 11;13:802362. doi: 10.3389/fgene.2022.802362 (PMC9709885; doi:10.3389/fgene.2022.802362)
Supplement: Supplementary file 1 [file Table1.DOCX]

**Supplementary Table 1.** Primers used for microsatellite marker analysis for chromosome 21.

| **Microsatellite marker** | **Chromosome location** | **Primer sequence (5′-3′)** | **Fluorescent label** |
| --- | --- | --- | --- |
| D21S411 | 21p11.1-p11.2 | F: ACATGTTAACATGCTATATCTGT | 6-FAM |
|  |  | R: GAACCATTATAAGTTGACCATC |  |
| D21S369 | 21q11.1 | F: ATGGCCTTGGCTAAATGCTG | 6-FAM |
|  |  | R: CTAAGCTGATATGGTAAGTACA |  |
| D21S120 | 21q11.1 | F: GTGTGTCTGCCATTTCTGGGTGTAG | VIC |
|  |  | R: GATCCTGGGACAAAGTAGTCTCTAA |  |
| D21S236 | 21q11.1 | F: CCCAAATAAAAAAGAGAACAG | VIC |
|  |  | R: CTAAAGAGGACTTCAGAGTAAGG |  |
| D21S408 | 21q11.1-q11.2 | F: AAGGTTGATTTCTACTGAGGC | 6-FAM |
|  |  | R: GGTGAGTTCTAATTCAATATCAC |  |
| D21S415 | 21q11.2 | F: CCTGATTTGTCTTTCATCTCG | 6-FAM |
|  |  | R: TGCCTGCTGTTGGACTTACT |  |
| D21S1414 | 21q21.1 | F: GGCACCCAGTAAAAAATTACT | VIC |
|  |  | R: CTGTCTGTCTGTCTGTCTATC |  |
| D21S1264 | 21q21.1 | F: GGCATAGAAGCCAAACATTT | VIC |
|  |  | R: CCAAACGAAATAGGAACAGCT |  |
| D21S1440 | 21q22.13 | F: GAGTTTGAAAATAAAGTGTTCTGC | 6-FAM |
|  |  | R: CCCCACCCCTTTTAGTTTTA |  |
| D21S2055 | 21q22.3 | F: AACAGAACCAATAGGCTATCTATC | VIC |
|  |  | R: TACAGTAAATCACTTGGTAGGAGA |  |
