# Supplementary material for: Case report: Molecular analysis of a 47,XY,+21/46,XX chimera using SNP microarray and review of literature
Source: Front Genet. 2022 Nov 11;13:802362. doi: 10.3389/fgene.2022.802362 (PMC9709885; doi:10.3389/fgene.2022.802362)
Supplement: Supplementary file 2 [file Presentation1.pptx]

## Slide 1
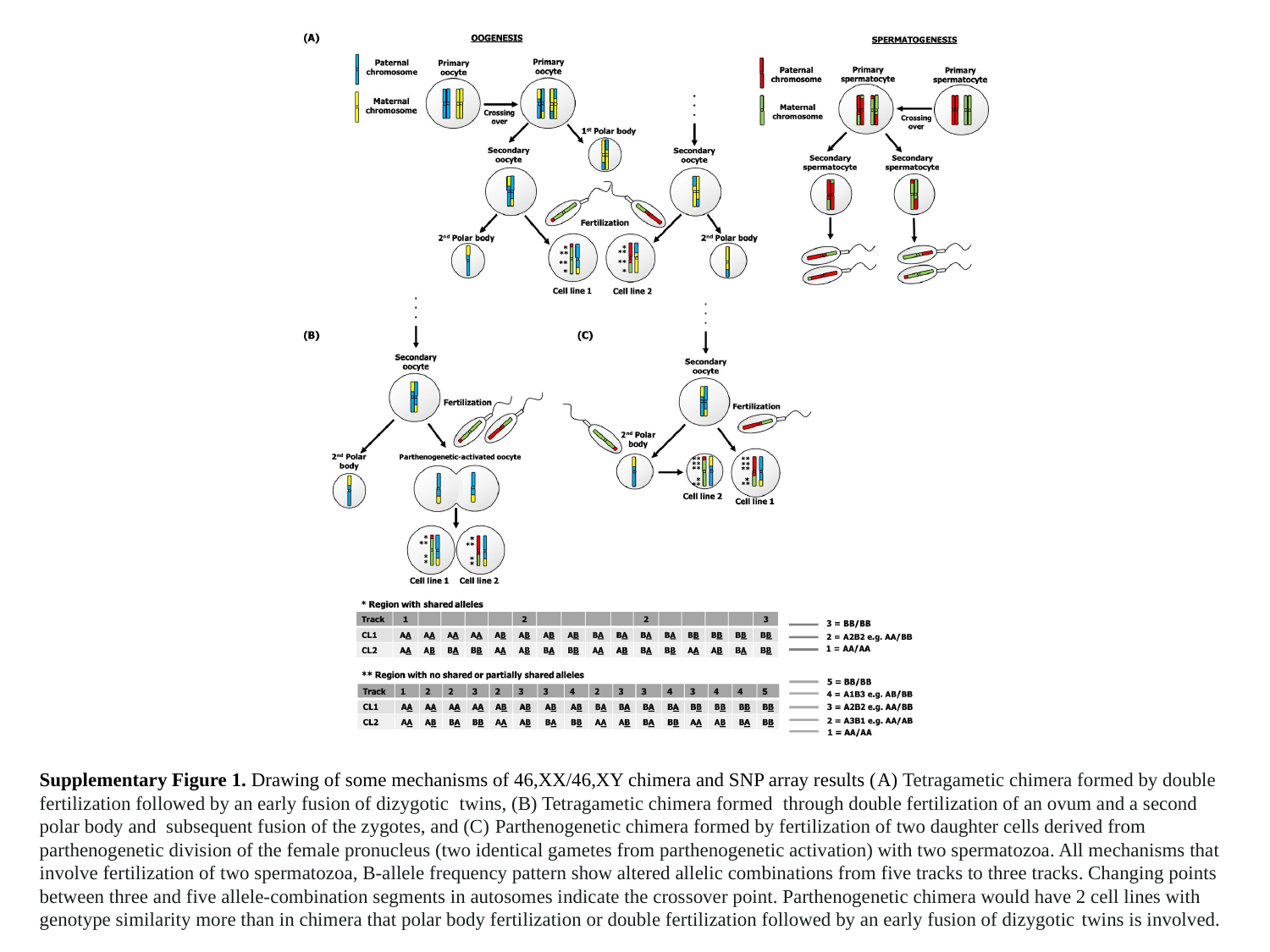

Supplementary Figure 1. Drawing of some mechanisms of 46,XX/46,XY chimera and SNP array results (A) Tetragametic chimera formed by double fertilization followed by an early fusion of dizygotic  twins, (B) Tetragametic chimera formed  through double fertilization of an ovum and a second polar body and subsequent fusion of the zygotes, and (C) Parthenogenetic chimera formed by fertilization of two daughter cells derived from parthenogenetic division of the female pronucleus (two identical gametes from parthenogenetic activation) with two spermatozoa. All mechanisms that involve fertilization of two spermatozoa, B-allele frequency pattern show altered allelic combinations from five tracks to three tracks. Changing points between three and five allele‐combination segments in autosomes indicate the crossover point. Parthenogenetic chimera would have 2 cell lines with genotype similarity more than in chimera that polar body fertilization or double fertilization followed by an early fusion of dizygotic twins is involved.
