# Supplementary material for: Case report: Molecular analysis of a 47,XY,+21/46,XX chimera using SNP microarray and review of literature
Source: Front Genet. 2022 Nov 11;13:802362. doi: 10.3389/fgene.2022.802362 (PMC9709885; doi:10.3389/fgene.2022.802362)
Supplement: Supplementary file 3 [file Table2.DOCX]

**Supplementary Table 2. Summary of STR analysis using human identification kit.** Double paternal contribution and single maternal contribution were observed for markers CSF1PO, D7S1517, TH01, D18S51, and D19S433. Single paternal contribution and double maternal contribution were observed for markers D3S1358 and D12S391. Analysis for markers on chromosome 21 and the remaining markers were inconclusive.

| **Genetic marker** | **Chromosome** | **Father** | **Mother** | **Patient** | **Patient’s Allelic ratio** |
| --- | --- | --- | --- | --- | --- |
| D2S1338 | 2q35 | 18, 23 | 19, 23 | 18, 19 | 0.53: 0.47 |
| TPOX | 2p25.3 | 8, 8 | 9, 11 | 8, 9 | 0.53: 0.47 |
| D3S1358 | 3p21.31 | 16, 16 | 17, 18 | 16, 17, 18 | 0.50: 0.07: 0.43 |
| FGA | 4q28 | 23, 24 | 22, 24 | 23, 24 | 0.53: 0.47 |
| CSF1PO | 5q33.1 | 12, 13 | 10, 11 | 12, 13, 10 | 0.41: 0.06: 0.53 |
| D5S818 | 5q23.2 | 11, 12 | 11, 12 | 11, 12 | 0.55: 0.45 |
| D7S1517 | 7q31.32 | 19, 22 | 25, 25 | 19, 22, 25 | 0.10: 0.42: 0.48 |
| D7S820 | 7q21.11 | 11, 11 | 11, 12 | 11, 11 | - |
| D8S639 | 8p22 | 24, 26 | 26, 27 | 24, 26, 27 | 0.06: 0.49: 0.45 |
| D8S1179 | 8q24.13 | 11, 14 | 14, 15 | 11, 14 | 0.07: 0.93 |
| TH01 | 11p15.5 | 6, 9 | 7, 8 | 6, 9, 7 | 0.44: 0.05: 0.51 |
| D12S391 | 12p13.2 | 17, 18 | 20, 24 | 17, 20, 24 | 0.62: 0.03: 0.35 |
| vWA | 12p13.31 | 16, 17 | 16, 17 | 16, 17 | 0.57: 0.43 |
| D13S317 | 13q31.1 | 8, 8 | 9, 10 | 8, 9 | 0.51: 0.49 |
| D16S539 | 16q24.1 | 10, 12 | 9, 9 | 12, 9 | 0.48: 0.52 |
| D18S51 | 18q21.33 | 13, 17 | 14, 16 | 13, 17, 14 | 0.10: 0.39: 0.51 |
| D19S433 | 19q12 | 13, 15.2 | 14, 14 | 13, 15.2, 14 | 0.11: 0.39: 0.50 |
| D21S11 | 21q21.1 | 29, 32.2 | 28, 32.2 | 32.2, 32.2 | - |
| D21S1437 | 21q21.1 | 14, 16 | 14, 15 | 14, 15 | 0.65: 0.35 |
| Amelogenin | Xp22.2, Yp11.2 | X, Y | X, X | X, Y | 0.91: 0.09 |
