# Supplementary material for: Case report: Molecular analysis of a 47,XY,+21/46,XX chimera using SNP microarray and review of literature
Source: Front Genet. 2022 Nov 11;13:802362. doi: 10.3389/fgene.2022.802362 (PMC9709885; doi:10.3389/fgene.2022.802362)
Supplement: Supplementary file 4 [file Table3.DOCX]

**Supplementary Table 3.** Summary of STR analysis of chromosome 21, X, and Y

| **STR marker** | **Chromosomal location** | **PCR product sizes (bp)** | | | **Patient’s allelic ratio** |
| --- | --- | --- | --- | --- | --- |
|  |  | **Father** | **Mother** | **Patient** |  |
| D21S1437 | 21q21.1 | 133, 141 | 133, 136 | 132, 136 | Inconclusive ratio (1.52) |
| D21S11 | 21q21.1 | 243, 257 | 239, 257 | 257, 257 | NA* |
| D21S1409 | 21q21.2 | NA | NA | 183, 196 | 1:1 |
| D21S1435 | 21q21.3 | 192, 192 | 183, 187 | 183, 192 | 1:1 |
| D21S1442 | 21q21.3 | 389, 393 | 369, 389 | 389, 393 | 1:1 |
| D21S1280 | 21q22.11 | NA | NA | 350, 354 | 1:1 |
| D21S1444 | 21q22.13 | 467, 471 | 467, 471 | 467, 467 | NA* |
| D21S2055 | 21q22.2 | NA | NA | 435, 451 | 1:1 |
| D21S1411 | 21q22.3 | 293, 305 | 301, 305 | 293, 301 | 1:1 |
| D21S411 | 21p11.1-p11.2 | 200, 200 | 200, 200 | 200, 200 | NA* |
| D21S369 | 21q11.1 | 174, 178 | 172, 178 | 172, 174 | 1:1 |
| D21S120 | 21q11.1 | 304, 313 | 304, 313 | 313, 313 | NA* |
| D21S236 | 21q11.1 | 122, 122 | 103, 122 | 122, 122 | NA* |
| D21S408 | 21q11.1-q11.2 | 162, 164 | 162, 164 | 162, 162 | NA* |
| D21S415 | 21q11.2 | 132, 136 | 132, 136 | 132, 136 | 1:1 |
| D21S1414 | 21q21.1 | 289, 299 | 285, 299 | 299, 299 | NA* |
| D21S1264 | 21q21.1 | 123, 127 | 119, 125 | 123, 125, 127 | 0.36: 1: 1 |
| D21S1440 | 21q22.13 | 154, 157 | 154, 160 | 154, 157, 160 | 1: 0.23: 1 |
| D21S2055 | 21q22.3 | 169, 189 | 152, 152 | 152, 169, 189 | 1: 1: 0.09 |
| DXS1187 | Xq26.2 | 151 | 139,147 | 147,151 | 1:1 |
| XHPRT | Xq26.2-q26.3 | 290 | 282,290 | 290,290 | NA* |
| DXS2390 | Xq27.1-q27.2 | 331 | 327,331 | 331,331 | NA* |
| DXYS267 | Xq21.31, Yp11.31 | 196,196 | 192,200 | 192,196 | 1:1 |
| DXYS218 | Xp22.33, Yp11.32 | 239,243 | 239,239 | 239,243 | 1:1 |
| AMELX | Xp22.2 | 104 | 104,104 | 104,104 | AMELX:AMELY 1:0.09 |
| AMELY | Yp11.2 | 109 | - | 109 |  |
| SRY | Yp11.31 | 237 | - | 237 | NA |
| ZFY, ZFX | Yp11.31, Xp22.11 | 161,164 | 164,164 | 164,164 | NA* |
| - | Xq13 | 202 | 202,202 | 202,202 | NA* |
| - | Xq21.1 | 137 | 137,137 | 137,137 | NA* |

*Cannot calculate allelic ratio due to homozygous markers
