# Supplementary material for: Case report: Molecular analysis of a 47,XY,+21/46,XX chimera using SNP microarray and review of literature
Source: Front Genet. 2022 Nov 11;13:802362. doi: 10.3389/fgene.2022.802362 (PMC9709885; doi:10.3389/fgene.2022.802362)
Supplement: Supplementary file 5 [file Table4.DOCX]

**Supplementary Table 4. SNP array trio analysis of “no-call” allele.** A total of 2513 SNP loci in the patient were categorized into no-call genotype. We excluded no-call genotype in the patient that no-call genotype also presented in either parent. Of these, 2496 SNP loci (99%) had homozygous (AA or BB) alleles contributed from the mother and heterozygous (AB) allele contributed from the father. Chromosome Y is not showed in the table because it must be contributed from the father.

| **Chromosome** | **Number of SNPs** | |
| --- | --- | --- |
|  | **Father = AB, Mother = AA or BB** | **Father = AA or BB, mother = AB** |
| 1 | 139 | 1 (rs17407336) |
| 2 | 12 | 1 (rs7564251) |
| 3 | 210 | 1 (rs2078116) |
| 4 | 56 | 0 |
| 5 | 116 | 1 (rs10462063) |
| 6 | 49 | 0 |
| 7 | 126 | 2 (rs2030925, rs219823) |
| 8 | 201 | 1 (rs7010112) |
| 9 | 101 | 0 |
| 10 | 81 | 0 |
| 11 | 191 | 1 (rs10751198) |
| 12 | 193 | 3 (rs11064121, rs12305483, rs708812) |
| 13 | 162 | 1 (rs778294) |
| 14 | 147 | 0 |
| 15 | 21 | 2 (rs580413, rs7174746) |
| 16 | 161 | 0 |
| 17 | 186 | 0 |
| 18 | 36 | 0 |
| 19 | 93 | 0 |
| 20 | 40 | 0 |
| 21 | 46 | 0 |
| 22 | 108 | 0 |
| X | 21 | 3 (rs1682037, rs5962008, rs6619061) |
| **Total** | **2496 (99.3%)** | **17 (0.7%)** |
